# A Microstrip Antenna Using I-Shaped Metamaterial Superstrate with Enhanced Gain for Multiband Wireless Systems

**DOI:** 10.3390/mi14020412

**Published:** 2023-02-09

**Authors:** Bukola Ajewole, Pradeep Kumar, Thomas Afullo

**Affiliations:** Discipline of Electrical, Electronic and Computer Engineering, University of Kwazulu-Natal, Durban 4041, South Africa

**Keywords:** microstrip patch antenna (MPA), metamaterial (MTM), permittivity, permeability, high gain, multi-band communications

## Abstract

This paper presents the design of a rectangular microstrip patch antenna (MPA) using the I-shaped metamaterial (MTM) superstrate. A seven × seven array of the I-shaped MTM unit cell is used as the superstrate to enhance the antenna performance. The antenna is fed by a microstrip feeding technique and a 50 Ω coaxial connector. An in-phase electric field area is created on the top layer of the superstrate to improve the performance of the antenna. The proposed I-shaped MTM-based rectangular MPA produces three operating frequencies at 6.18 GHz, 9.65 GHz, and 11.45 GHz. The gain values of the proposed antenna at 6.18 GHz, 9.65 GHz and 11.45 GHz are 4.19 dBi, 2.4 dBi, and 5.68 dBi, respectively. The obtained bandwidth at frequencies 6.18 GHz, 9.65 GHz and 11.45 GHz are 240 MHz (3.88%), 850 MHz (8.8%), and 1010 MHz (8.82%), respectively. The design and simulation of the antenna are done using the Computer Simulation Technology (CST) studio suite and MATLAB. The proposed I-shaped MTM-based rectangular MPA is fabricated on a low-cost FR-4 substrate and measured using the Agilent 8719ET network analyzer. The proposed antenna has an overall dimension of 70 × 70 × 1.6 mm^3^. A significant improvement in the gain of the antenna up to 74.28% is achieved. The obtained results confirm that the proposed multiband antenna has a high gain, and enhancement in bandwidth and radiation efficiency. These properties make the proposed antenna suitable for the multiband wireless communications systems such as Wi-Fi devices, radar systems, short- and long-range tracking systems, etc.

## 1. Introduction

Microstrip patch antennas (MPAs) have been widely used in various wireless and mobile communication systems since 2002 after the Federal Communication Commission (FCC) opened its use to civilians [1]. An antenna is widely acknowledged to be a critical component in communication systems. To keep up with the development trend, a high-performance ultra-wideband antenna with a compact structure must be designed. In comparison to other conventional antennas, due to its high efficiency, wide bandwidth, miniaturized size, low spectral density, affordability, and flexibility, the ultra-wideband antenna has received much attention recently. Apart from the numerous benefits of MPAs, there are a few drawbacks, including low gain, low efficiency, low power handling, narrow impedance, and excessive radiation from feeds [2,3,4,5]. Numerous researchers have conducted extensive research on metamaterial structures to mitigate these constraints by enhancing the MPA performance in terms of bandwidth, gain, directivity, and efficiency.

MTMs have been discovered to be excellent candidates for improving antenna characteristics. MTMs are artificially designed structures with negative permeability and permittivity properties at specific resonant frequencies [6,7]. Tunable MTMs can be designed using liquid crystals. The tunability in liquid crystals-based metamaterials can be achieved employing various strategies such as external voltage, power tunability of liquid crystals’ parameters, etc. [8,9,10,11]. The MTM surfaces help to reduce surface waves as well as provide in-phase reflections, which improves the MPA performance in terms of gain, directivity, efficiency, and bandwidth [12]. The gain of the MPA was enhanced by using a cylindrical EBG structure, as described in [13]. The antenna resonates at 2.6 GHz with a gain enhancement of 2.9 dBi. Weng et al. [14] developed a new design of an MTM-inspired MPA for multiband functionality. The MTM structure was used as the antenna substrate instead of the conventional substrate material. The designed antenna produced a gain of 8.2 dB at 2.77 GHz. Three different types of UWB antennas with triple-notched bands were proposed and studied by Zhang et al. [15]. The proposed design includes a planar circular patch and split ring resonators (SRRs) integrated into the MPA’s feedline. Pattnaik et al. [16] proposed the rectangular MPA loaded with multiple SRRs. The resonant frequency of the MPA was reduced from 14.08 GHz to 6.15 GHz when the proposed MTM was loaded. Singh et al. [17] investigated the effect of different structural parameters on the bandwidth and resonant frequency of an improved MTM structure. It has been found that reducing the size of the patch increases the resonant frequency and bandwidth, making it suitable for X-band applications. Tang et al. [18] designed an MPA with triband notches. The MPA has SRR structures, and filters interference from wireless local area networks (WLANs) and WiMAX. The MPA provides a broad bandwidth range of 3.03 GHz to 11.4 GHz with triband notches.

Patel et al. [19] proposed and designed an MTM superstrate embedded MPA. The metallic integration of MTM in the superstrate consists of an SRR, which improves antenna performance. The bandwidth of the proposed MPA increased up to 60%. A triangular-shaped MTM-inspired triband MPA with a dielectric substrate of Roger RT/Duroid 5870 was proposed by Alam et al. [20]. The described MPA has an optimum gain of 4.06 dB with the frequency bands at 3.3–3.6 GHz, 5.15–5.35 GHz, and 5.725–5.825 GHz. Therefore, the proposed MPA can be used for both the lower and higher bands of DCS 1800, Bluetooth, WiMAX, and WLAN. Rajkumar et al. proposed an MTM-inspired compact open SRR MPA for multiband applications [21]. When the open split rings are utilized as the radiating element in this design, the size of the MPA is reduced when compared to a ring of identical size. The proposed MPA is built and operates at frequencies of 2.4 GHz, 4.1 GHz, and 5.2 GHz. A single-band planar resonant MPA inspired by phi-shaped slotted MTM and a square MPA were proposed by Saravanan et al. [22]. Using MTM and photonic crystal as superstrate, the gain of the MSA was enhanced. The dimensions of the antenna are 61.25 × 61.25 mm^2^.

Hasan et al. [23] proposed and designed a low-profile SRR-loaded MTM-inspired MPA. The MPA was fabricated on an FR-4 dielectric material with the dimensions of 30 × 31 mm^2^. Two MTM unit cells aligned with one another were incorporated into the antenna patch. The proposed MPA has resonance frequencies of 2.47 GHz and 3.62 GHz, a minimum gain of 0.88 dBi, and a maximum gain of 2.25 dBi. The MPA’s radiation and gain were improved by using substrate-integrated waveguide (SIW) technology [24]. The MPA’s radiation and gain were improved. Inserting a column of metallic connectors between the MeTM unit cells improves the antenna’s performance greatly. With a maximum gain of 5.80 dBi, a frequency range of 3–4.80 GHz was obtained. In [25], an MTM structure was loaded with a slotted double negative material (DNG) over Rogers R0430B dielectric substrate. The antenna operated at 4.34 GHz with a maximum gain of 7.14 dBi. A multi-layered SRR was staked over the patch antenna [26]. A multiband antenna with metamaterial was proposed in [27] for WiMax/WLAN applications. A dual-band compact SRR shaped antenna was proposed and investigated in [28]. The antenna has frequency bands of 1.75–2 GHz and 3.01–4.18 GHz with the realized gain of 1.5 dBi and 2.05 dBi, respectively. A multi-band microstrip patch antenna MTM was proposed by Ashyap et al. [29]. The metamaterial structure was used as the radiating patch. The proposed antenna performed well at 0.36 THz, 0.49 THz, 0.69 THz, 0.87 THz and 1.04 THz.

For wireless communication, Ajewole et al. [30] designed a square SRR MTM-inspired MPA. The proposed MTM unit cell is made up of two rings, with the outer ring having four split gaps and the inner ring having a single cut. The antenna gain and directivity were increased from 4.04 dBi to 5.3 dBi and 5.8 dBi to 6.7 dBi, respectively. A quintuple circular SRR was designed to enhance the performance of a rectangular MPA by G.Al. Duhni [31]. The SRR-loaded MPA produced five resonance frequencies at 8.16 GHz, 9.08 GHz, 10.06 GHz, 10.73 GHz, and 11.34 GHz. 

This research work builds on our initial design in [30] and incorporates a significantly improved design. Considering the reduction in the operating frequency band reported in [16], low gain in some antennas such as in [21,23,28], and low gain enhancement reported in [22,30], this work aims to provide a robust approach to tackle these limitations. The uniqueness of this work lies in the design of the proposed I-shaped MTM array superstrate utilized by the proposed antenna for multi-band applications. The I-shaped MTM array is designed using the I-shaped MTM cell [32]. In this work, the design, simulation, fabrication, and measurement of the rectangular MPA using an I-shaped MTM array superstrate are presented. The proposed model of the I-shaped MTM array superstrate is integrated with the rectangular MPA, and this shows a significant improvement in terms of gain, bandwidth, and efficiency. A maximum gain enhancement of 74.28% is achieved. The rest of the paper is organized as follows: the design and geometry of the rectangular MPA, I-shaped MTM array and the rectangular MSA with the I-shaped superstrate are presented in Section 2. Section 3 presents the results and discussion. The conclusion of the research work is presented in Section 4.

## 2. Design and Geometry of the I-Shaped MTM based MPA

This section describes the design process for the proposed I-shaped MTM-based MPA. The I-shaped MTM structure is analyzed and used to improve the performance of the designed I-shaped MTM-based MPA.

### 2.1. Geometry of the I-Shaped MTM Superstrate

**Figure 1:**
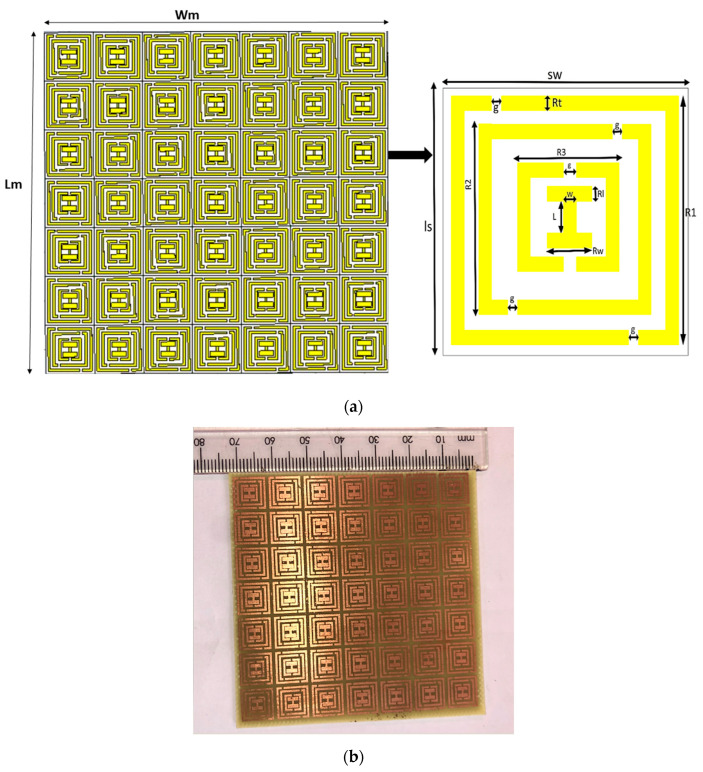
7 × 7 I-shaped MTM array: (**a**) geometrical configuration, (**b**) fabricated structure.

The geometry and fabricated structure of the 7 × 7 I-shaped MTM array is illustrated in Figure 1. The MTM array is printed on the FR-4 substrate with a dielectric constant of 4.3 and a loss tangent (tanδ) of 0.025. The thickness of the substrate (sh) and the thickness of the annealed copper *(hc)* used for the split ring resonator are 1.6 mm and 0.035 mm, respectively. The primary function of splits in the ring resonators is to ensure that the inductance and capacitance interact with one another to determine the operating frequency. The unit cell’s total optimum size is 10 × 10 × 1.6 mm^3^ $\left(0.2\lambda_{0}\times 0.2\lambda_{0}\times 0.03\lambda_{0}\right)$ [32]. The dimensional parameters of the 7 × 7 I-shaped MTM array are presented in Table 1.

The effective parameters of the MTM can be determined by carefully placing the structure between two ports of the waveguides, with an electromagnetic wave with magnetic and electric fields along the y- and x-axes, respectively. This implies that the first port transmits the reflecting signal, while the second port serves as the receiving end. The effective medium ratio is proportional to the unit cell dimension, and the wavelength must be less than the working wavelength. The reflection and transmission coefficient can be given as [33,34]:

(1) $$S_{11}=\frac{\left(1-\Gamma^{2}\right)Z}{1-\Gamma^{2}Z^{2}}$$

(2) $$S21=\frac{\left(1-Z^{2}\right)\Gamma }{1-\Gamma^{2}Z^{2}}$$

where S11, S21 and Z represent the reflection coefficient, transmission coefficient and impedance, respectively. By using Equations (2) and (3), the intermediate parameters $V_{1}$ and $V_{2}$ can be defined as [33,34]:

(3) $$V_{1}=S11+S21$$

(4) $$V_{2}=S21-S11$$

**Figure 2:**
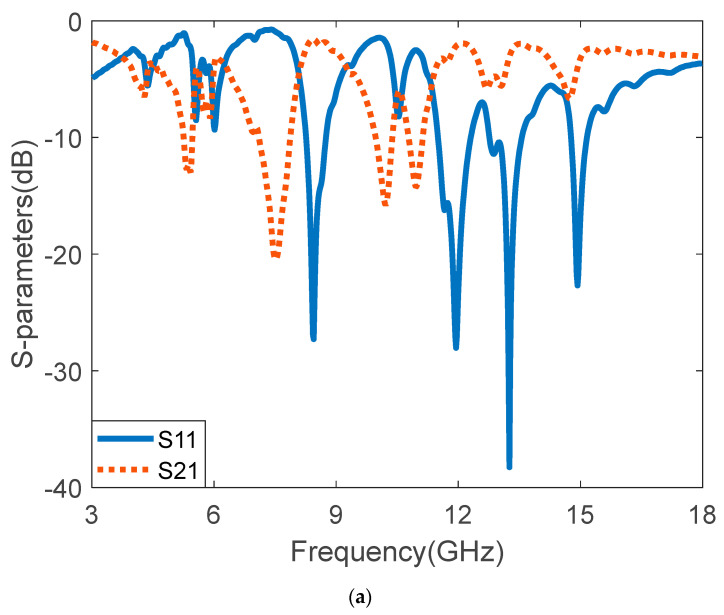

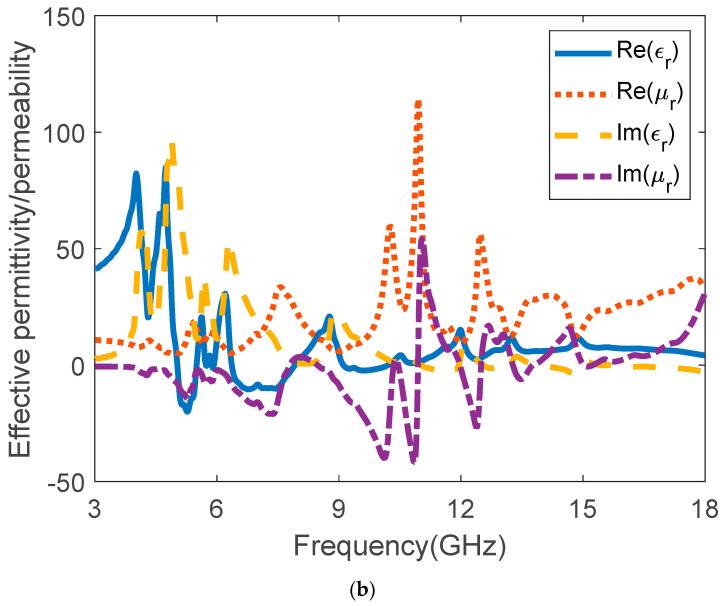
I-shaped 7 × 7 MTM array analysis: (**a**) S-parameters, (**b**) effective electromagnetic parameters (permittivity and permeability).

The Nicolson-Ross-Weir (NRW) method is adopted to determine the electric permittivity $\left(\varepsilon_{r}\right)$ and the magnetic permeability ($\mu_{r})$ of the MTM as given below [33,34]:

(5) $$\varepsilon_{r}=\frac{2}{j\pi fS_{h}}\left(\frac{1-V_{2}}{1+V_{1}}\right)$$

(6) $$\mu_{r}=\frac{2}{j\pi fS_{h}}\left(\frac{1-V_{1}}{1+V_{2}}\right)$$

where f and $S_{h}$ denote the operating frequency and the height of the substrate, respectively. The scattering parameters of the MTM unit cell and array can be analyzed by using the above equations. The S-parameters and the effective parameters of the proposed 7 × 7 I-shaped MTM array superstrate are shown in Figure 2. The simulated S-parameters for the 7 × 7 I-shaped MTM array are presented in Figure 2a. The S-parameters show resonance frequencies at 4.31, 5.45, 6.07, 7.42, 8.29, 10.85, 12.38, and 15.01 GHz in the C, X, and Ku- bands. The real and imaginary values of the effective permeability and permittivity are shown in Figure 2b. The structure produces permittivity characteristics for the frequency ranges 4.3, 5.1–5.5, 5.9–8.2, and 9.1–18 GHz. The I-shaped MTM array exhibits multi-band properties.

### 2.2. Design and Geometry of Rectangular MPA Using I-Shaped MTM Superstrate

**Figure 3:**
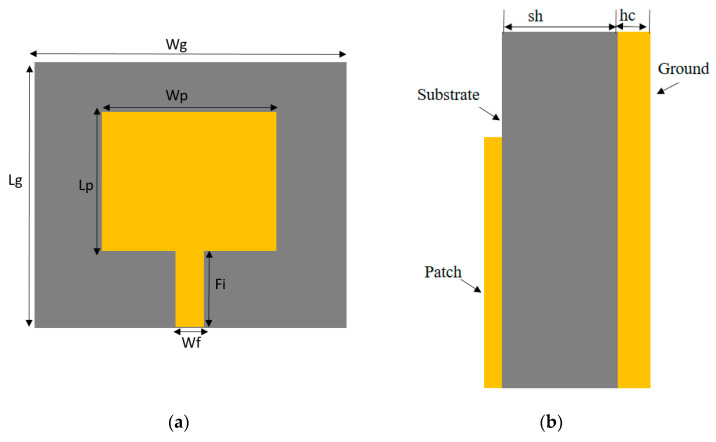

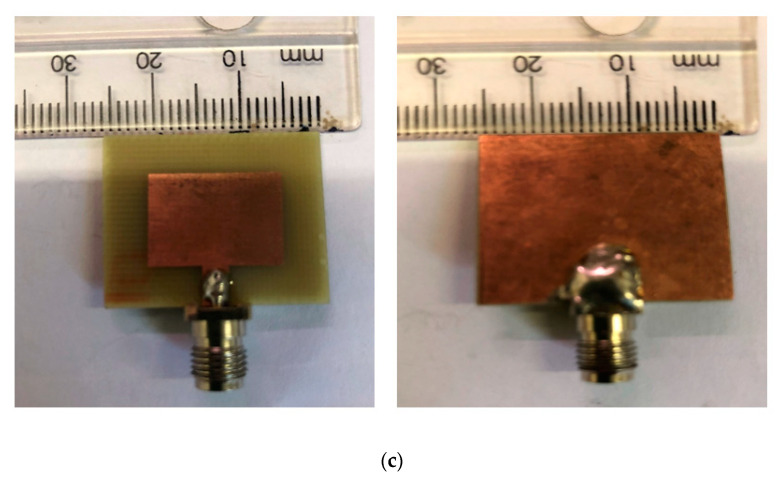
Rectangular MPA: (**a**) top view (**b**) side view (**c**) fabricated top and back view.

The top view of the geometrical structure and the side view of the rectangular MPA are depicted in Figure 3a and Figure 3b, respectively. The rectangular MPA is designed and fabricated on the FR-4 dielectric substrate. The top view and bottom view of the fabricated antenna are shown in Figure 3c. The SMA coaxial connector of 50 Ω along with the microstrip line are used to feed the antenna.

The geometric parameters of the rectangular MPA, such as width and length, are calculated by using the transmission line model equations which can be found in [35,36]. The size of the ground plane and patch of the antenna are $0.50\lambda_{0}\times 0.42\lambda_{0}$ and $0.31\lambda_{0}\times 0.23\lambda_{0}$, respectively ($\lambda_{0}$ = free space wavelength). The rectangular MPA configuration and simulation are done using the finite integration technique-based electromagnetic CST simulator. The dimensions of the rectangular MPA are presented in Table 2.

**Figure 4:**
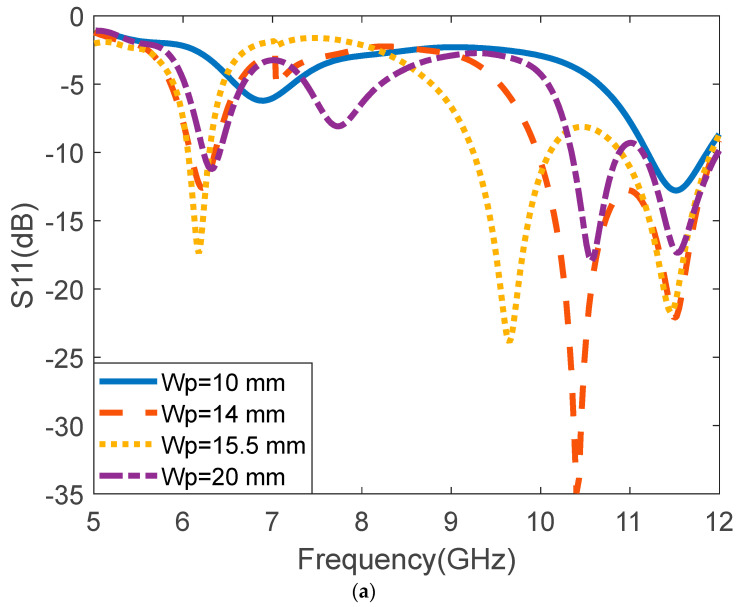

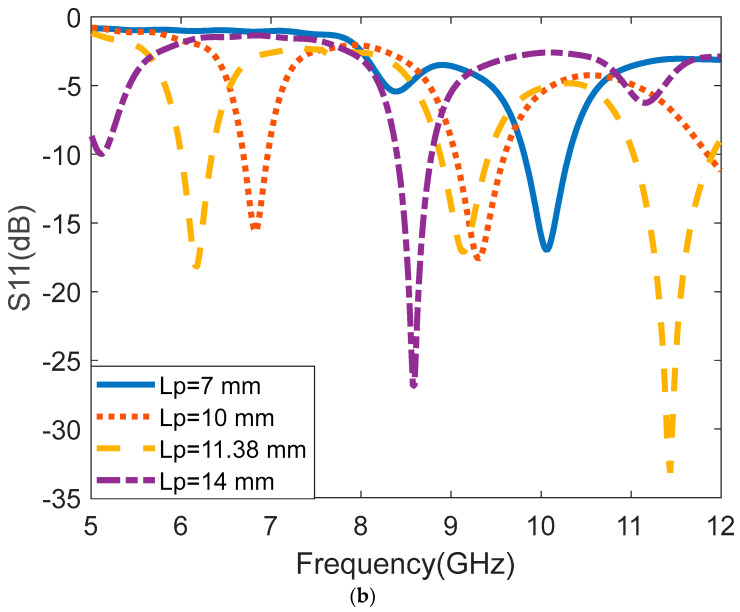
Effect of variation of Wp and Lp on the reflection coefficient, (**a**) S11 for different values of Wp, (**b**) S11 for different values of Lp.

The effect of the width of the patch ($W_{p}$) and length of the patch ($L_{p}$) on the reflection coefficient is presented in Figure 4a and Figure 4b, respectively. The $W_{p}$ is varied from 10 mm to 20 mm. It was observed that at 10 mm the patch produced one resonance frequency at 11.5 GHz with a return loss of −12.78 dB. At 14 mm, two resonance frequencies were observed at 6.03 GHz and 10.4 GHz with a reflection coefficient of −13.4 dB and −34.97 dB, respectively. When the patch dimension was increased to 20 mm it produced three frequencies at 6.22 GHz, 10.6 GHz, and 11.5 GHz. The reflection coefficient at 6.22 GHz, 10.6 GHz, and 11.5 GHz are −11.1 dB, −18 dB, and −17.3 dB, respectively. It can be observed that the rectangular MPA has better performance at 15.3 mm with three operating frequencies at 6.18 GHz, 9.09 GHz, and 11.48 GHz, with a reflection coefficient of −17.9 dB, −17.6 dB, and −34.4 dB, respectively. The presented antenna is suitable for C/X-band high gain multi-band wireless communication applications. The operating frequencies 9.09 GHz and 11.48 GHz are due to the higher order modes. Form Figure 4b, it can be observed that the resonant frequency decreases upon increasing the value of $L_{p}$. The analysis of the resonant frequencies for different modes is carried out using the Equation (7) [37]:

(7) $$f_{r\left(m,n,p\right)}=\frac{c}{2\sqrt{\varepsilon_{reff}}}\sqrt{{\left(\frac{m}{sh}\right)}^{2}+{\left(\frac{n}{L_{p}}\right)}^{2}+{\left(\frac{p}{W_{p}}\right)}^{2}}$$

where $f_{r\left(m,n,p\right)}$ is the resonant frequency of the $TM_{mnp}$ mode. For the computation of the resonant frequencies, the variation of the simulated values of the dielectric constant with the frequency is considered, as given in Table 3. The computed and simulated resonant frequencies of the rectangular MPA are given in Table 4. From Table 4, it can be observed that the analytical resonant frequencies and simulated resonant frequencies are closely matched. Hence, using the higher mode resonant frequencies analysis and optimization in CST microwave studio, the presented antenna can be designed for the other specified frequency bands.

**Figure 5:**
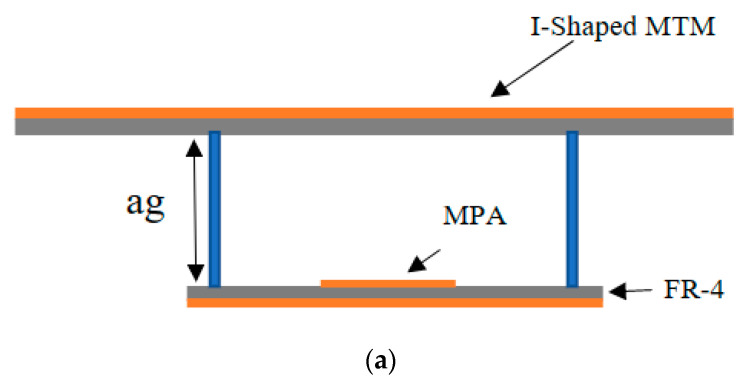

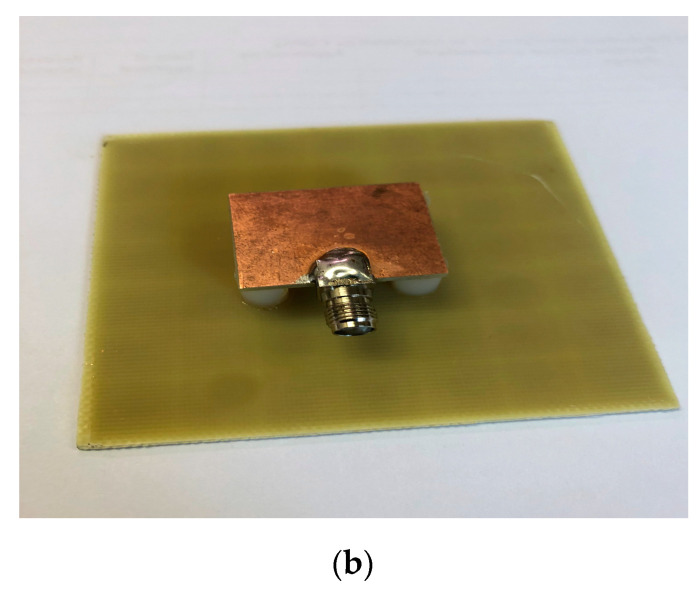
Proposed I-shaped MTM-based rectangular MPA (**a**) Geometrical configuration (**b**) Fabricated prototype.

The geometrical configuration of the designed I-shaped MTM-based rectangular MPA is shown in Figure 5a. The final structure is obtained by using the superstrate designed with the 7 × 7 I-shaped MTM array integrated with the rectangular MPA with an overall dimension of 70×70 mm^2^ ($1.4\lambda_{0}\times 1.4\lambda_{0}$). The fabricated prototype of the proposed antenna is shown in Figure 5b. The term (“I-shaped MTM-based rectangular MPA”) and (“proposed antenna”) are interchangeably used throughout this paper.

## 3. Result and Discussion

**Figure 6:**
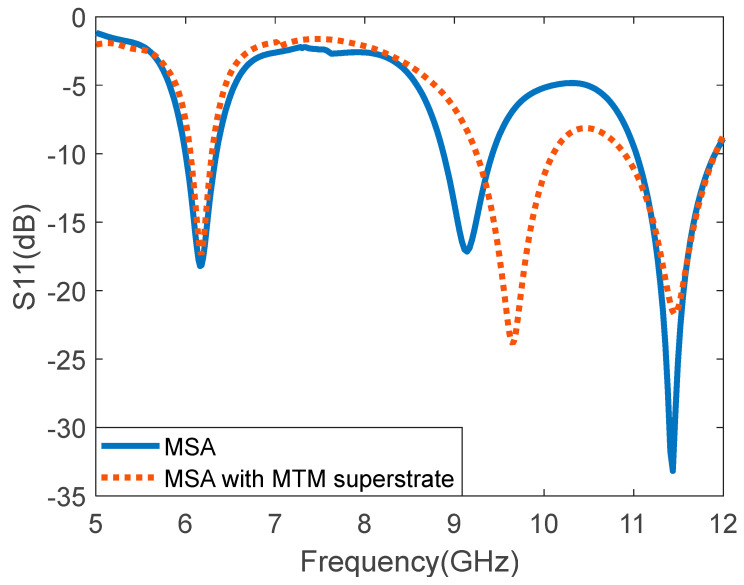
Simulated reflection coefficient (S11) of the rectangular MPA and I-shaped MTM array-based rectangular MPA.

The simulated reflection coefficient S11 (dB) for the rectangular MPA and I-shaped MTM-based MPA is illustrated in Figure 6. It is observed that the rectangular MPA resonates at 6.18 GHz, 9.14 GHz, and 11.48 GHz with a bandwidth of 330 MHz, 700 MHz, and 800 MHz, respectively. When the I-shaped MTM is integrated with the rectangular MPA, the resonant frequencies are at 6.18 GHz, 9.65 GHz, 11.5 GHz with a bandwidth of 240 MHz (3.88%), 850 MHz (8.8%), and 1010 MHz (8.82%), respectively. The bandwidth of the first resonant frequency became narrower when the rectangular MPA was loaded with the superstrate. However, a significant increase in the bandwidth, 21.43% for the second band and 26.25% for the third band, is observed.

**Figure 7:**
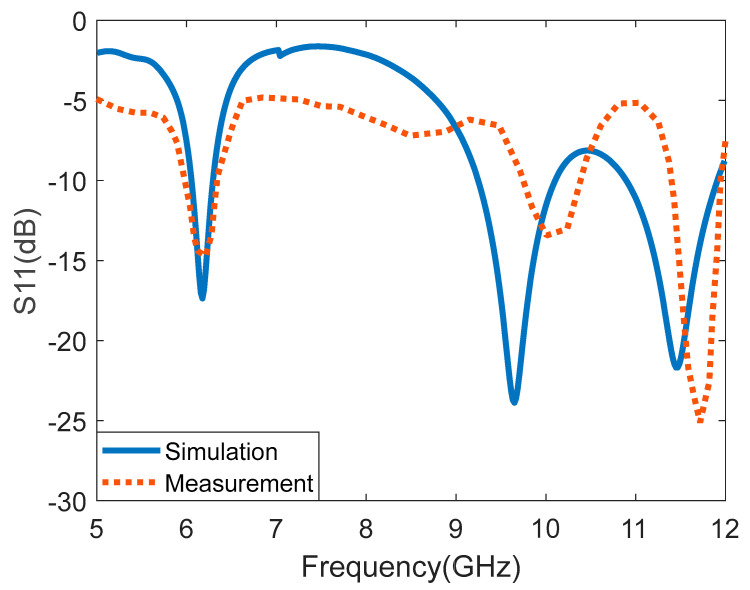
Simulated and measured reflection coefficient (S11) of the antenna.

**Figure 8:**
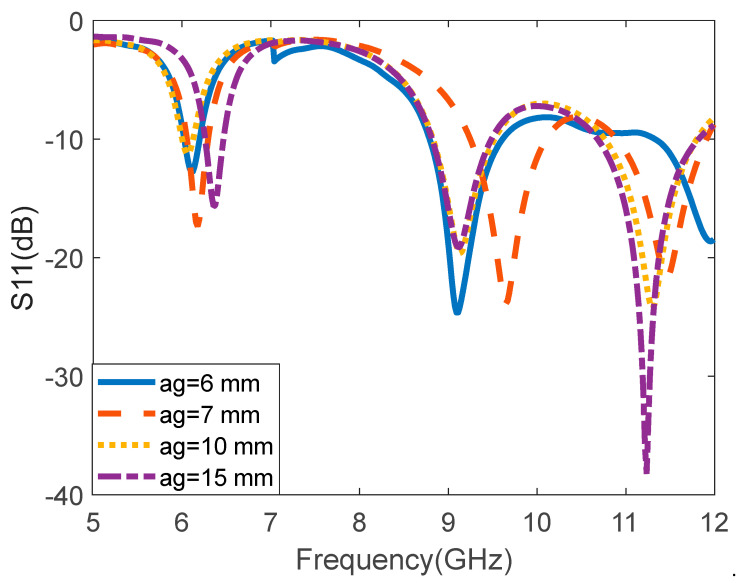
Reflection coefficient (S11) of the antenna for different values of ag.

The Agilent 8719ET network analyzer was used to measure the proposed I-shaped MTM-based rectangular MPA. Figure 7 depicts the simulated and measured S11 (dB) of the I-shaped MTM-based rectangular MPA. The measured resonant frequencies are 6.18 GHz, 9.94 GHz, and 11.7 GHz. A slight disparity can be observed between the simulated and measured S-parameter, which can be attributed to fabrication tolerance, variation of material specification, calibration of the network analyzer, and impedance from the connector soldering. Figure 8 depicts the reflection coefficient of the proposed antenna at various heights (ag). The plastic spacers separate the superstrate and rectangular MPA. The spacers have no significant impact on the antenna’s performance. The distance between the rectangular MPA and the I-shaped MTM superstrate is varied from 6 mm to 15 mm. The reflection coefficient characteristics for the four values of ag, i.e., ag = 6 mm, ag = 7 mm, ag = 10 mm, and ag = 15 mm are presented in Figure 8. It is observed that for the first and second bands, ag = 7 mm provides the minimum reflection coefficient along with the wide bandwidth. However, for the third band, ag = 15 mm provides the minimum reflection coefficient with almost same bandwidth as ag = 7 mm. Considering the overall performance for all the frequency bands, for ag = 7 mm, the antenna gives the optimum performance.

**Figure 9:**
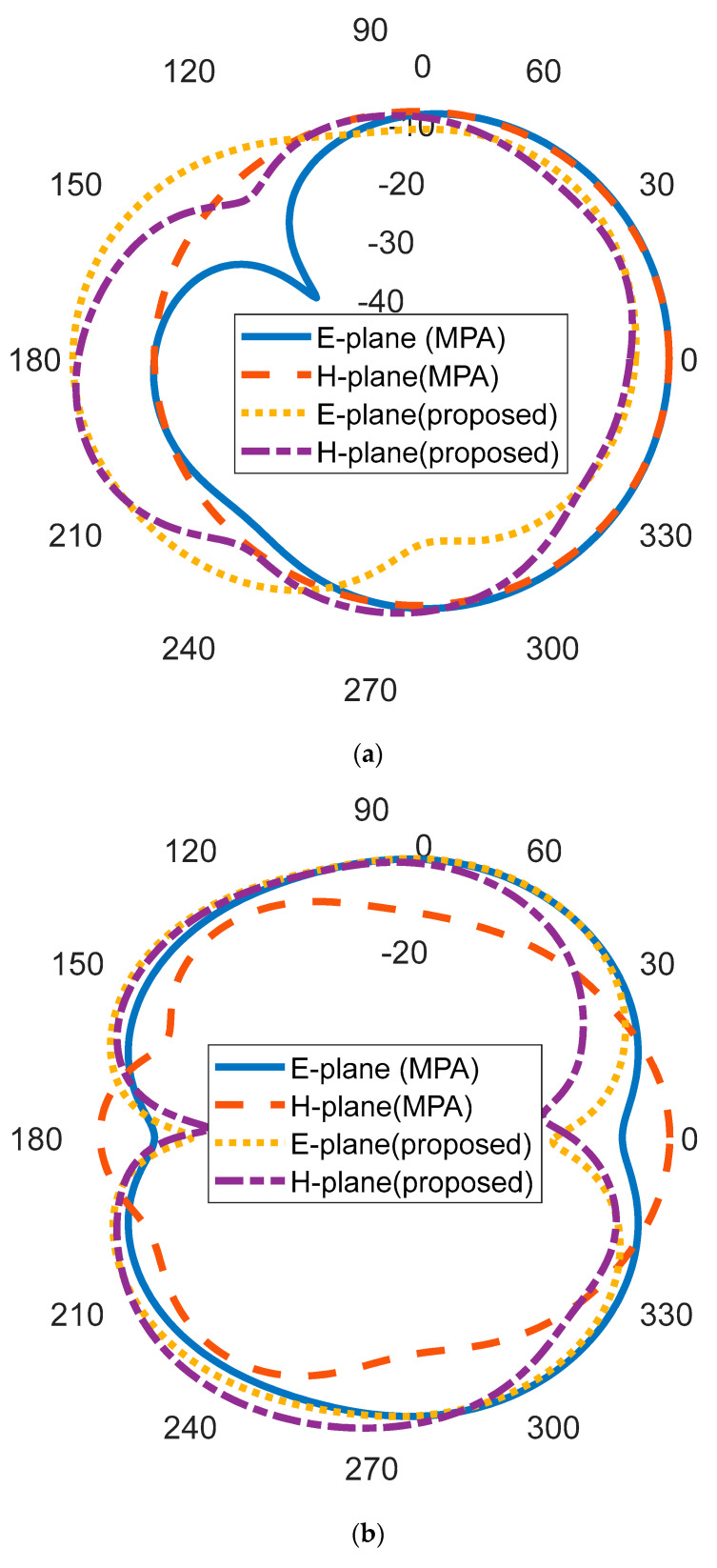

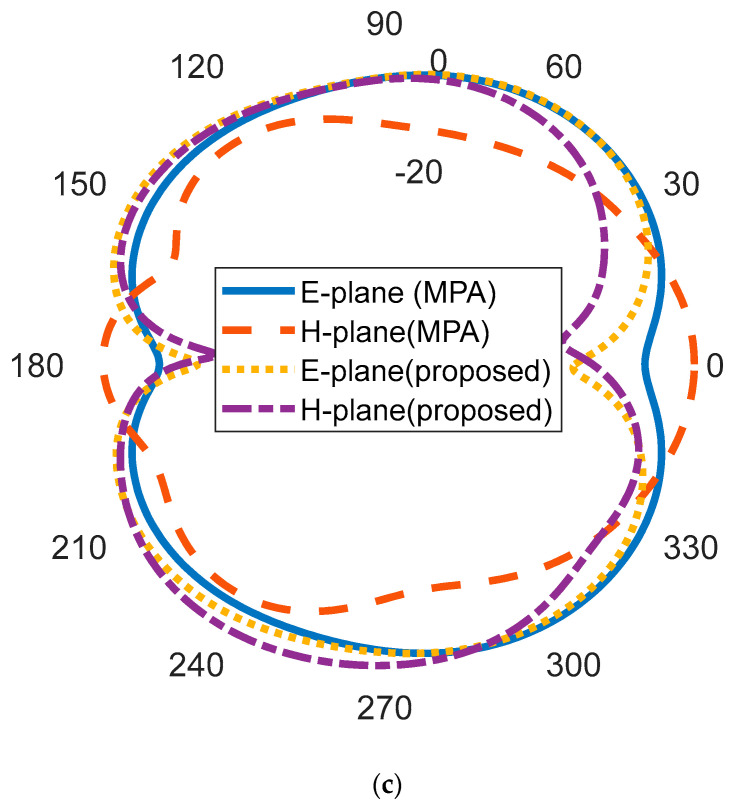
Radiation patterns of the rectangular MPA and proposed antenna at: (**a**) 6.18 GHz (**b**) 9.65 GHz (**c**) 11.5 GHz.

The normalized simulated 2-D radiation patterns of the rectangular MPA and the proposed antenna at three operating frequencies are shown in Figure 9. The resonant frequency 6.18 GHz in E- plane (phi = 0^0^) and H- plane (phi = 90^0^) shows a broadside radiation pattern. In Figure 9b, an omnidirectional radiation pattern is observed at 9.65 GHz. In Figure 9c, the antenna shows a dipole-like pattern at E-plane and an omnidirectional pattern at the H-plane. 

**Figure 10:**
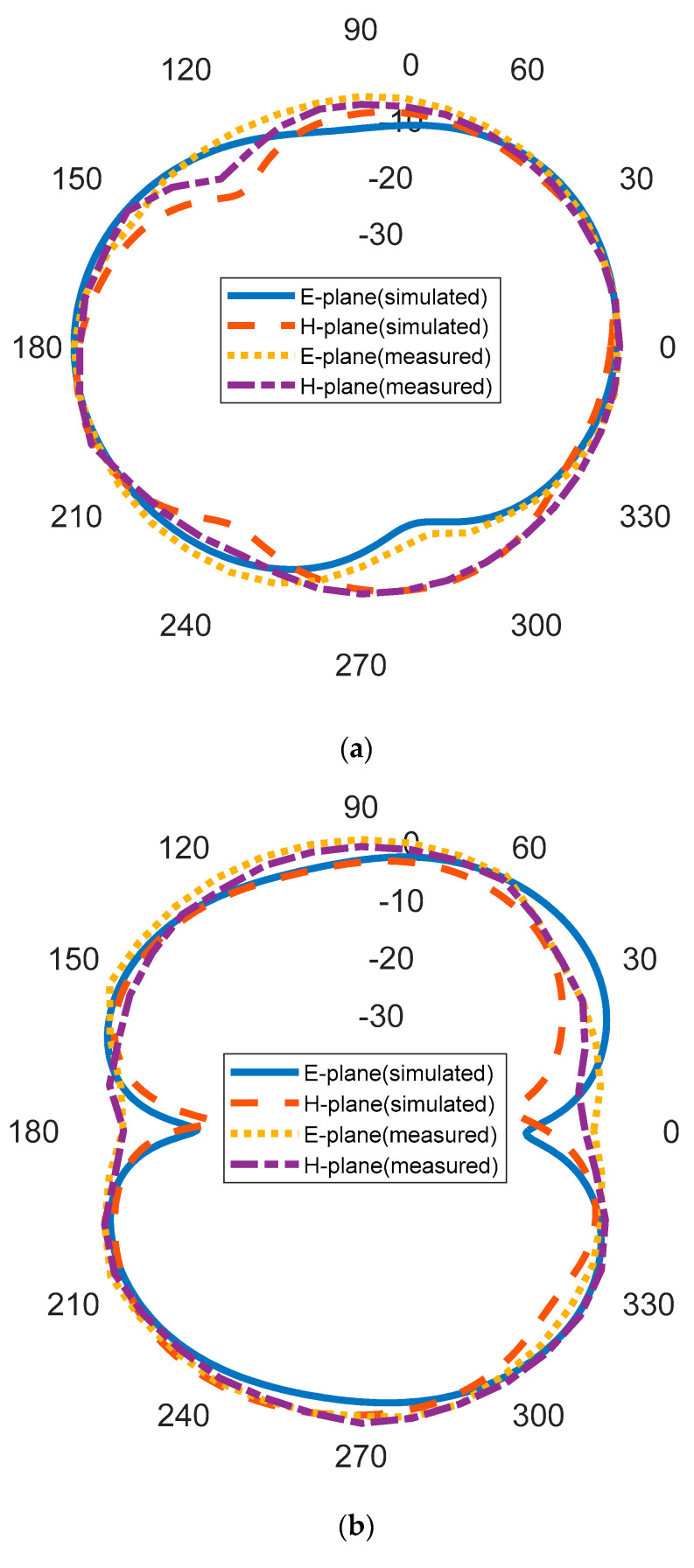

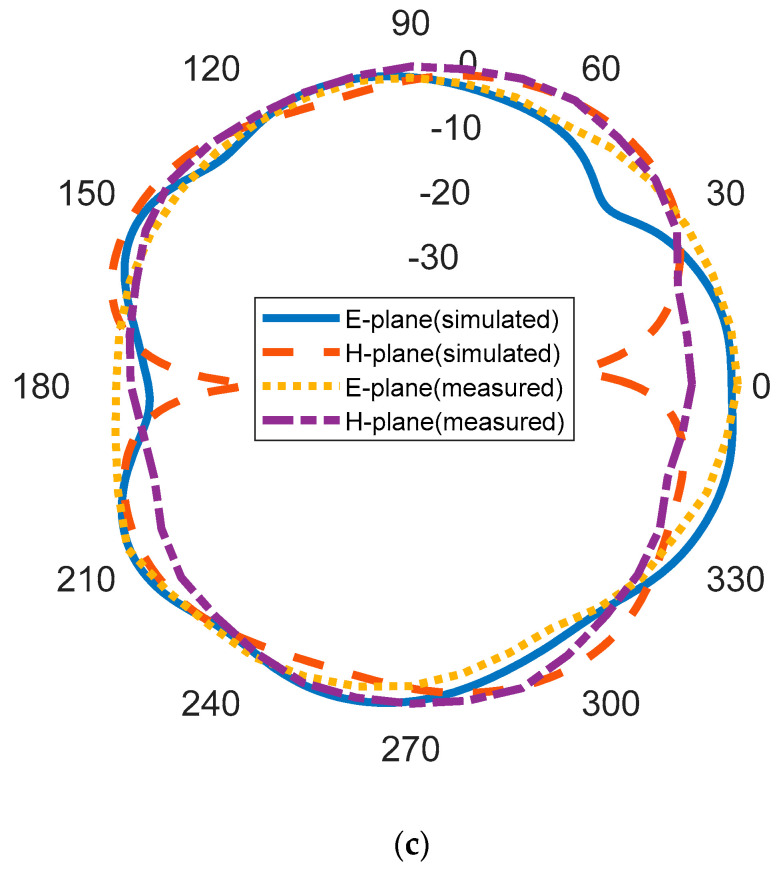
Radiation patterns of the proposed antenna: (**a**) at 6.18 GHz (**b**) at 9.65 GHz (**c**) at 11.5 GHz.

The realized gain for the rectangular MPA and the proposed antenna is presented in Table 5. It can be observed that there is significant improvement in the gain of the antenna integrated with the I-shaped MTM array. The gain of the rectangular MPA increased significantly at all the frequencies for the antenna integrated with the I-shaped MTM array superstrate. At 6.18 GHz, the gain increased from 2 dBi to 4.18 dBi, while at 9.14 GHz a gain of 0.09 dBi was obtained by the rectangular MPA. As the I-shaped MTM array is integrated with the rectangular MPA, a gain of 2.39 dBi is achieved. A gain increase from 3.22 dBi to 5.63 dBi was experienced at 11.48 GHz. The gain enhancement in % for various frequencies is given in Table 5. It can be observed that the gain enhancement of 74.28% at 11.48 GHz is achieved. The simulated and measured radiation patterns of the proposed I-shaped MTM-based rectangular MPA at 6.18 GHz, 9.09 GHz, and 11.5 GHz are shown in Figure 10a, Figure 10b, and Figure 10c, respectively. It can be observed that the simulated and measured patterns are in good agreement.

The angular 3 dB beamwidth, main lobe direction, total and radiating efficiency, maximum gain, and directivity of the proposed antenna are presented in Table 6. It can be observed that as the operating frequency of the antenna increases, the total and radiating efficiency of the antenna decreases. The comparison of the existing works with the I-shaped MTM-based rectangular MPA is presented in Table 7. From this comparison, it can be observed that the proposed low-cost antenna provides multiband operation, high gain, and high gain enhancement. Various antenna parameters confirm the suitability of the proposed antenna for multiband C/X-band wireless systems such as Wi-Fi devices, radar systems, and short- and long-range tracking systems.

## 4. Conclusions

A multiband rectangular MPA integrated with an I-shaped MTM superstrate array has been presented. The proposed antenna utilized a seven × seven I-shaped MTM array to improve the performance of the antenna. The rectangular MPA and the I-shaped MTM array are designed, fabricated, and etched on an FR4 substrate. The proposed antenna produces three resonance frequencies at 6.18 GHz, 9.65 GHz, and 11.45 GHz. The gain of the antenna has been improved significantly. A gain enhancement up to 74.28% has been achieved. The proposed low-cost antenna has a high gain and enhancement in both bandwidth and radiation efficiency. These properties make the proposed antenna suitable for multiband wireless communications systems such as Wi-Fi devices, radar systems, short- and long-range tracking systems, etc.

## Figures and Tables

**Table 1:** Dimensional parameters of the 7 × 7 array.

Parameter	Dimension (mm)	Parameter	Dimension (mm)
Wm	70	R3	2.8
Lm	70	Rt	0.5
Sw	10	Rl	2
Ls	10	W	0.5
Rw	1.25	sh	1.6
R1	9	ch	0.035
R2	4	g	0.5

**Table 2:** Dimensional parameters of the rectangular MPA.

Parameter	Dimension (mm)	Parameter	Dimension (mm)
$W_{g}$	24.96	Fi	4.36
$L_{g}$	20.98	sh	1.6
$W_{p}$	15.4	hc	0.035
$L_{p}$	11.38	ag	7
Wf	3.01		

**Table 3:** Dielectric constant and loss tangent at various frequencies.

Parameter	Value (at 5.45 GHz)	Value (at 6.07 GHz)	Value (at 7.27 GHz)	Value (at 8.29 GHz)	Value (at 10.85 GHz)	Value (at 12.38 GHz)
Dielectric constant	4.34	4.33	4.32	4.31	4.29	4.28
Loss tangent	0.0235	0.0239	0.0245	0.0248	0.0250	0.0249

**Table 4:** Computed and simulated resonant frequencies of various $TM_{mnp}$ modes for the rectangular MPA.

S. No.	Mode	Resonant Frequency (Computed)	Resonant Frequency (Simulated)
1.	$TM_{010}$	6.043 GHz	6.18 GHz
2.	$TM_{002}$	8.950 GHz	9.09 GHz
3.	$TM_{020}$	12.138 GHz	11.48 GHz

**Table 5:** Realized gain of the rectangular MPA and the proposed antenna.

S. No.	Frequency	Gain of the Rectangular MPA	Gain of the Proposed Antenna	Gain of the Rectangular MPA (Absolute Value)	Gain of the Proposed Antenna (Absolute Value)	Gain Enhancement
1.	6 GHz	1.91 dBi	3.42 dBi	1.55	2.2	41.94%
2.	6.18 GHz	2 dBi	4.18 dBi	1.58	2.62	65.82%
3.	9.14 GHz	0.09 dBi	2.39 dBi	1.02	1.73	69.6%
4.	11.48 GHz	3.22 dBi	5.63 dBi	2.1	3.66	74.28%
5.	12 GHz	2.91 dB	5.25 dBi	1.95	3.35	71.79%

**Table 6:** Radiation parameters of the I-shaped MTM-based rectangular MPA.

Antenna Parameters	Rectangular MPA			Proposed Antenna		
	6.18 GHz	9.14 GHz	11.44 GHz	6.18 GHz	9.65 GHz	11.5 GHz
Angular beamwidth (3dB) (phi = 0°)	91.1	152.0	64.5	66	57	81.1
Angular beamwidth (3dB) (phi = 90°)	98.0	74.8	63.1	43.9	93	56.3
Main lobe direction (phi = 0°)	0.0	35	0.0	180	49	146
Main lobe direction (phi = 90°)	30.8	42	54	168	127	51
Radiating efficiency (dB)	−3.670	−5.929	−5.042	−3.894	−3.196	−2.176
Total efficiency (dB)	−4.074	−9.530	−5.352	−4.059	−3.199	−2.168
Maximum gain (dB)	2.000	0.098	3.229	4.192	2.388	5.679
Maximum directivity (dB)	5.671	6.020	8.260	8.005	5.697	7.073

**Table 7:** Comparison of the proposed antenna with the related literature.

Ref.	Dimension	Resonance Frequency	Substrate	Gain (dBi)	Gain Enhancement	Remarks
Boutayeb and Denidni [13]	180 mm ×180 mm	2.6 GHz	Taconic	9.33	94.7%	Single-band
Weng et al. [14]	165 mm × 165 mm	1.66 GHz	$\varepsilon_{r}=2.65$	5.8	--	Not a low-cost FR-4
		2.02 GHz		0.8		
		2.40 GHz		4.2		
		2.48 GHz		3.6		
		2.77 GHz		8.2		
Patel and Kosta [19]	50 mm × --	3.51 GHz	FR-4	--	--	Gain not reported
		4.86 GHz				
		7.8 GHz				
Alam et al. [20]	48 mm × 48 mm	1.9 GHz	Rogers RT5870	1.64	--	Low gain
		2.45 GHz		2.07		
		5 GHz		4.06		
Rajkumar and Usha Kiran [21]	27.75 mm × 16.08 mm	2.4 GHz	FR-4	0.37	--	Low gain
		4.1 GHz		1.61		
		5.2 GHz		1.88		
Saravanan and Umarani [22]	61.25 mm × 61.25 mm	2.4 GHz	FR4 Metamaterial	6.56	30.17%	Single-band, Low gain enhancement
Hasan et al. [23]	31 mm × 30 mm	2.47 GHz	FR-4	1.88	--	Dual-band, low gain
		3.62 GHz		1.35		
Pandya et al. [26]	45 mm × 35 mm	1.13 GHz	FR-4	3.73	--	Low gain for two bands
		2.47 GHz		6.18		
		2.74 GHz		1.35		
Patel et al. [28]	56 mm × 56 mm	1.75–2.0 GHz	FR-4	1.5	--	Dual-band, low gain
		3.01–4.18 GHz		2.05		
Ajewole et al. [30]	25 mm × 21.4 mm	5.8 GHz	FR-4	5.27, 4.04	32.67%	Single band, low gain enhancement
Rao et al. [38]	14 mm × 12 mm	28 GHz	Roger RT/Duroid 6006 and Roger RT/ Duroid 5880	6.36 (5)	37.02%	Single-band, low gain enhancement
Pragati et al. [39]	46 mm × 32 mm	2.45 GHz	FR4	2.76 (2.4)	8.62%	Low gain, Low gain enhancement
		3.5 GHz		−7.4 (−8.2)	20%	
		4.65 GHz		3.68 (1.4)	68.84%	
Kaur et al. [40]	70 mm × 70 mm	2.53 GHz	FR4	3.64	--	Dual band, low gain
		5.77 GHz		3.84		
Kucukcan and Kaya [41]	50 mm × 50 mm	2.41 GHz	FR4	0.29	--	Low gain
		5.8 GHz		1.17		
Proposed antenna	70 mm × 70 mm	6.18 GHz	FR-4	4.19	65.82%	Triple-band, low-cost FR4, high gain, high gain enhancement
		9.65 GHz		2.4	69.6%	
		11.5 GHz		5.68	74.28%	

-- not applicable/reported.

## Data Availability

Not applicable.
